# Spigelian Hernia: A Rare Ventral Hernia

**DOI:** 10.7759/cureus.55209

**Published:** 2024-02-29

**Authors:** Yulia Shtanko, Juliet Galtes, Wilmer Mata

**Affiliations:** 1 College of Medicine, Florida International University, Herbert Wertheim College of Medicine, Miami, USA; 2 General Surgery, Baptist Health South Florida, Miami, USA

**Keywords:** general and laparoscopic surgery, laproscopic hernia repair, hernias, risk factors, laparoscopic repair, ventral hernia, spigelian hernia, general surgery

## Abstract

Ventral hernias occur when abdominal contents or the peritoneum displace through a defect in the abdominal wall. Among these, spigelian hernias are an exceptionally rare subtype, representing 0.12% to 2% of all ventral hernias. This case study focuses on an 86-year-old female presenting with a ventral hernia, notably a spigelian hernia, lacking common predisposing factors. The study emphasizes the use of laparoscopic techniques for repair, aiming to offer insights into managing this infrequent hernia type and aiding clinical decision-making. Due to its low incidence and challenging diagnosis and identification, reports such as ours detailing both the clinical course and the operative steps can assist others in their clinical decision-making.

## Introduction

Spigelian hernias are an exceedingly rare type of ventral hernia. The defect in the case of a ventral hernia occurs between the transversus abdominis, extending from the cartilage of the ninth rib to the pubic tubercle, and the internal oblique aponeurosis [1]. Anatomically, the semilunar line is the border of these structures. The spigelian fascia transitions to the posterior sheath of the rectus abdominis at the arcuate line, and this transition creates an innate weakness known as the spigelian belt. In this region, there is also increased abdominal circumference and pressure. With these factors considered, the spigelian hernia is most likely to form in that location [2].

Spigelian hernias have an incidence rate of only 0.12% to 2% and are most commonly observed in females who are often in the fifth or sixth decade of life; they can be congenital or acquired [1]. In addition, patients generally have other comorbidities that have led to elevated intra-abdominal pressures. The elevated intra-abdominal pressures would weaken the fascial layers of the abdomen, increasing the risk of a ventral hernia forming. These comorbidities include chronic obstructive pulmonary disease (COPD), ascites, pregnancy, Ehlers-Danlos syndrome, peritoneal dialysis, or obesity [3,4,5].

Although a patient presenting with abdominal pain and these comorbidities may raise suspicion for a hernia, clinical diagnosis may be challenging. Many patients with spigelian hernias do not have a distinct palpable mass. Importantly, requesting the patient to perform the Valsalva maneuver while standing may help identify the mass. Regardless, up to 50% of spigelian hernias will not be identified by a physical exam alone [1]. CT and ultrasound can be other modalities used for diagnosis. CT is the most reliable tool for diagnosis.

## Case presentation

An 86-year-old female with a past medical history of hypertension, hypothyroidism, hyperlipidemia, insomnia, and polymyalgia rheumatica presented to the emergency department (ED) with 3 days of gradually worsening abdominal pain, nausea, and vomiting. 

In the ED, the patient was found to have an elevated blood pressure of 223/75, regular heart rate of 85 bpm, afebrile oral temperature of 36.7 °C, and oxygen saturation of 98% on room air. Physical exam was significant for left lower abdominal tenderness and dry mucous membranes. Initial labs were unremarkable, with the exception of elevated BUN, mildly elevated lactic acid, and leukocytosis (Table 1).

**Table 1:** Pre-operative laboratory values hemoglobin (HGB), hematocrit (HCT), blood urea nitrogen (BUN), white blood cell (WBC), pro-brain natriuretic peptide (pro-BNP) *For an adult woman

Laboratory Study	Lab Value	Reference Range
HGB	15.1 g/dL	11.6 - 15 g/dL *
HCT	45.8%	36% - 48% *
BUN	28 mg/dL	6 - 24 mg/dL
WBC	16.86 × 10^9/L	4.5 to 11.0 × 10^9^/L
Lactic Acid	3 mmol/L	<2 mmol/L
Pro-BNP	711 pg/mL	<450 pg/mL

**Figure 1:**
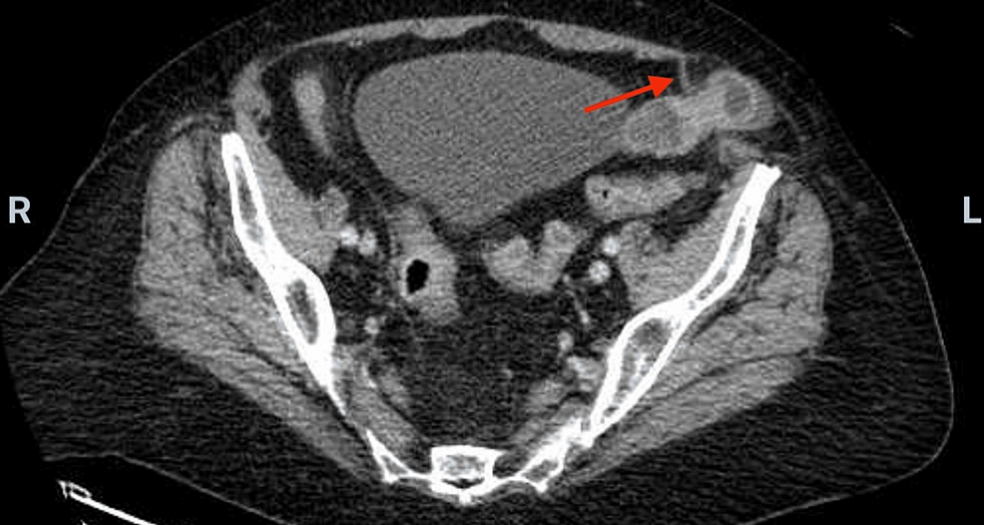
Abdominal CT scan with IV contrast The red arrow indicates the spigelian hernia, sized at less than 2 cm

An abdominal CT scan revealed a spigelian hernia containing a loop of small bowel at the level of the left lower quadrant (Figure 1).

The scan also showed a distended stomach and small bowel obstruction, likely secondary to bowel entrapment within the hernia. For this reason, she was admitted to the inpatient medical unit for further management. Upon admission, the patient was treated with Intravenous (IV) Pepcid 20 mg daily, IV Zofran 4 mg as needed for nausea, and IV hydralazine 10 mg for blood pressure control. Additionally, a nasogastric (NG) tube was placed for stomach decompression, continuous intravenous normal saline fluids were started for hydration, the patient was kept on Nothing Per Oral (NPO) status, and a surgical consult was placed. 

The surgical team noted that the NG tube drained dark content, the patient was not passing flatus, and there was severe pain on palpation of the left lower quadrant. Subsequently, she was scheduled for a laparoscopic-assisted left spicula hernia repair with mesh the following day. During the surgery, an incision was created just over the spigelian hernia, and tissues were dissected all the way down until the hernia sac was visible. The sac was then excised and a Ventralex™ mesh (Becton Dickinson) measuring 6 cm was placed over the defect. Prolene sutures were used to close the fascia in a figure-of-eight and the skin was closed with 4-0 Monocryl in a subcuticular fashion.

Post-operatively, the patient showed improvement in her vital signs, lab values, and pain. She was no longer hypertensive and her leukocytosis improved (Table 2).

**Table 2:** Post-operative laboratory values hemoglobin (HGB), hematocrit (HCT), white blood cell (WBC) *For an adult woman

Laboratory Study	Lab Value	Reference Range
HGB	11.5 g/dL	11.6 - 15 g/dL *
HCT	35.5 %	36% - 48% *
WBC	12.93 10^9/L	4.5 to 11.0 × 10^9^/L

Two days after surgery, the patient was tolerating food well, she was stooling and reported that her nausea, vomiting, and abdominal pain resolved. She was discharged home with outpatient follow-up in two weeks. 

## Discussion

Spigelian hernias draw attention for their rarity and challenge in recognition and diagnosis. In this case study, we present an 86-year-old female who was diagnosed with a spigelian hernia and highlight her presenting symptoms and clinical course.

Spigelian hernias are rare defects that develop between the transversus abdominus and the internal oblique aponeurosis [1]. Patients commonly present with abdominal pain and rarely exhibit a palpable mass. In our case, our patient presented with three days of ongoing abdominal pain, vomiting, and nausea. CT scan showed small bowel within the ventral defect, likely causing obstruction and related symptoms. An abdominal mass was never appreciated on a physical exam, however, sharp lower left quadrant pain was always noted. This emphasizes the importance of a CT scan in the proper diagnosis of a spigelian hernia. Spigelian hernias can become an emergency due to their high risk of incarceration and strangulation; therefore, a prompt CT scan and diagnosis, and operative repair via open, laparoscopic, or surgical techniques is required [1].

The open surgical technique can be done through a fascial or mesh closure. Initially, a paramedial or transverse incision is made and proceeds posteriorly until a hernia sac is identified and isolated. The hernia may be easily reduced, but omentectomy or bowel resection may be required depending on the case, and a mesh is placed. The closure of the fascia can be done with either absorbable or non-absorbable sutures [6,7].

Compared to open surgical techniques, laparoscopic techniques have been shown to have lower morbidity and length of stay [8]. There are two commonly accepted methods for laparoscopic correction including intraperitoneal onlay mesh repair or intraperitoneal repair. Similar to an open technique, the intraperitoneal method includes reduction of the hernia contents, adhesiolysis, and a placement of synthetic mesh over the defect. Extraperitoneal repair varies in that the peritoneum remains intact and is not entered. Robotic correction is similar to the intraperitoneal approach. However, robotic correction has limited data on its safety and effectiveness [9].

It is also important to note that spigelian hernias can occur in patients with little to no predisposing risk factors. For example, our patient did not have comorbidities associated with hernia development, such as COPD, ascites, Ehlers-Danlos syndrome, peritoneal dialysis, or obesity [3,4,5]. Despite this, she was a case that contributed to the 0.12% to 2% incidence rate of spigelian hernias [1]. 

Complications following robotic repair of Spigelian hernias occur in 12% of cases [10]. These complications encompass hematomas, seromas, abdominal viscera injury, hernia recurrence, and infections. Infections can affect the operative site or the mesh. Proper mesh selection is an important factor in preventing rates of post-operative complications. In our case, a Ventralex™ Hernia Patch, composed of self-expanding polypropylene and ePTFE patch was used. This is a permanent synthetic mesh, which is more susceptible to infection than absorbable synthetic meshes. Despite this, permanent synthetic meshes are associated with a lower rate of hernia recurrence than the latter. 

Although rare and difficult to diagnose, swift treatment is needed due to their high risk of incarceration and strangulation. In our case, small bowel was seen within the spigelian hernia and the patient required NG tube suctioning while awaiting a surgical evaluation. She experienced a favorable clinical course due to efficient radiologic diagnosis and surgical intervention with laparoscopy. 

## Conclusions

Despite its infrequent occurrence, a spigelian hernia is an important and possible diagnosis in the setting of abdominal pain. Predisposing risk factors to development are not always present and the diagnosis should be considered if symptoms support it. Symptoms include nausea, vomiting, and abdominal pain. Regardless of physical exam and symptoms, spigelian hernias are difficult to diagnose clinically and a CT scan is the most reliable tool for diagnosis. 

To prevent complications of small bowel incarceration and strangulation, it is important to consult Surgery for further management. A commonly practiced surgical technique is the laparoscopic resection of the hernia and defect correction with mesh. With proper surgical interventions, patients are expected to improve and confirm resolution of symptoms.
